# Symptoms of posttraumatic stress, anxiety, and depression, along with their associated factors, among Eritrean refugees in Dabat town, northwest Ethiopia, 2023

**DOI:** 10.1186/s40359-024-01554-7

**Published:** 2024-02-07

**Authors:** Mihret Melese, Wudneh Simegn, Dereje Esubalew, Liknaw Workie Limenh, Wondim Ayenew, Gashaw Sisay Chanie, Abdulwase Mohammed Seid, Alemante Tafese Beyna, Melese Legesse Mitku, Asefa Kebad Mengesha, Yibeltal Yismaw Gela

**Affiliations:** 1 Department of Human Physiology, School of Medicine, College of Medicine and Health Science, University of Gondar, Gondar, Ethiopia; 2 Department of Social and Administrative Pharmacy, School of Pharmacy, College of Medicine and Health Sciences, University of Gondar, Gondar, Ethiopia; 3 Department of Human Physiology, College of Medicine and Health Sciences, Ambo University, Ambo, Ethiopia; 4 Department of Pharmaceutics, School of Pharmacy, College of Medicine and Health Sciences, University of Gondar, Gondar, Ethiopia; 5 Department of Clinical Pharmacy, School of Pharmacy, College of Medicine and Health Sciences, University of Gondar, Gondar, Ethiopia; 6 Department of Pharmacology, School of Pharmacy, College of Medicine and Health Sciences, University of Gondar, Gondar, Ethiopia

**Keywords:** Depression, Anxiety, Posttraumatic stresses disorder, Eritrean refuges, Ethiopia

## Abstract

**Background:**

Refugee populations are forcibly displaced from their homes as a consequence of natural disasters and armed conflicts. Eritreans, initially displaced to the Maiayni camp within the Tigray region, have faced further relocation to Dabat town due to the conflict between the Tigray People Liberation Front (TPLF) and Ethiopian government forces. Subsequently, another conflict has arisen between the Amhara Popular Force (Fano) and Ethiopian government forces in Dabat town, disrupting its stability. These collective challenges in the new environment may contribute to the development of symptoms such as posttraumatic stress disorder (PTSD), anxiety, and depression. Currently, there is a lack of available data on these symptoms and their associated variables in Dabat Town. Thus, the objective of this study was to assess the prevalence of PTSD, anxiety, and depression symptoms, along with associated factors, among Eritrean refugees in Dabat town, northwest Ethiopia. This will provide significant evidence for developing and implementing mental health intervention strategies that specifically address the particular difficulties faced by refugees.

**Method:**

A community-based cross-sectional study was carried out from July 25 to September 30, 2023, in the Eritrean refugee camp in Dabat town. A systematic random sampling method was employed to select a total of 399 Eritrean refugees with 100 response rate. Data were collected using the standard validated Depression, Anxiety, and Stress Scale (DASS-21) questionnaire, which included socio-demographic characteristics. Summary statistics such as frequency and proportion were utilized to present the data in tables and figures. Binary logistic regression was employed to identify associated factors, and variables with a *p*-value (*p* ≤ 0.05) were considered statistically significant factors.

**Result:**

The findings of this study indicated that 45% (95% CI: 35.6-48.23), 33.6% (95% CI: 31.66–37.45), and 37.3% (95% CI: 35.56–40.34) of the participants had symptoms of depression, anxiety, and PTSD, respectively. Sex, age, employment status, lack of food or water, experience of torture or beating, and imprisonment emerged as statistically significant predictors of depression. Employment status, murder of family or friends, rape or sexual abuse, torture or beating, and lack of housing or shelter were statistically significantly associated with anxiety. PTSD was found to be significantly associated with sex, length of stay at the refugee camp, lack of housing, shelter, food, or water, experience of rape or sexual abuse, abduction, employment status, and murder of family or friends.

**Conclusions and recommendation:**

The results of this study revealed that more than one-third of Eritreans living in the refugee camp in Dabat town had symptoms of PTSD, anxiety, and depression. This prevalence is higher than the previously reported studies. Various factors, including age, gender, monthly income, unemployment, experiences of rape or sexual abuse, witnessing the murder of family or friends, being torched or beaten, imprisonment, and deprivation of basic needs such as food, shelter, and water, were identified as contributors to the development of depression, anxiety, and PTSD. This research underscores the need for both governmental and non-governmental organizations to secure the provision of essential necessities such as food, clean water, shelter, clothing, and education. This study also suggested that Eritrean refugees be legally protected from rape, sexual abuse, arson, detention without cause, and kidnapping. Moreover, the study calls for health service providers to develop a mental health intervention plan and implement strategies to deliver mental health services at healthcare facilities for Eritrean refugees in the Dabat town Eritrean refugee camp.

## Introduction

Refugee populations are forcibly displaced from their homes as a consequence of natural disasters and armed conflicts [1–4]. A significant burden of psychiatric morbidity falls on populations exposed to the effects of war and forced migration, particularly those suffering from disorders like PTSD, anxiety, and depression [5–9]. Refugees face a higher likelihood of encountering acts of extreme violence, becoming targets of terrorist attacks, experiencing abduction and torture, enduring family separation, and undergoing forced migration. These circumstances can exacerbate symptoms related to stress, anxiety, and depression [10–13]. In 2023, data from the UN High Commissioner for Human Rights reported more than 18,000 civilian victims, with 7,031 fatalities and 11,327 injuries arising from the conflict between Russia and Ukraine [14]. The Russia-Ukraine war has resulted in a significant rise in the number of refugees leaving Ukraine for neighboring countries, accounting for approximately 2,871,519 of the deaths and injuries [15]. Furthermore, within the country, around 5,914,000 individuals have been internally displaced since November [16].

The prevalence of mental disorders among forcibly displaced populations shows variability, ranging from 2 to 88% for posttraumatic stress disorder (PTSD), 5 to 81% for depression, and 1 to 90% for generalized anxiety disorder [17]. In 2017, worldwide, anxiety disorders affected 260 million individuals, and 300 million people were affected by depression, leading to economic consequences totaling at least $1 trillion (USD) in annual lost productivity [18].

A meta-analysis carried out in 2009 on populations exposed to conflict and refugees indicated a depression prevalence of 30.8% [19]. In a similar systematic study carried out in 2019, 8,176 Syrian refugees who had been resettled reported having anxiety symptoms at a rate of 26% and 40% of depression [20]. In the Gaza Strip’s refugee camps, 23.9% of Palestinians were found to be suffering from PTSD [21]. The prevalence of depression, anxiety, and stress differs among various refugee camps in Africa. In Southern Sudan, the documented prevalence of depression was 49.9% [22]; while, in Uganda, the rates were 32% for Rwandan refugees and 48.1% for Somali refugees, respectively [23]. In the Maiayni refugee camp in the Tigray region of Ethiopia, the prevalence of depression among Eritreans was documented at 37.8% [24].

A number of factors, such as advanced age, unemployment, gender, a higher number of potentially traumatic experiences, low socioeconomic status, substance use disorders, the nature of the trauma, witnessing someone being killed or seriously injured, and socio-demographic characteristics like low socioeconomic status and marital status, can lead to the development of symptoms of depression, anxiety, and PTSD [14, 25–30].

Eritreans, initially displaced to the Maiayni camp within the Tigray region, have faced further relocation to Dabat town due to the conflict between the Tigray People Liberation Front (TPLF) and Ethiopian government forces. Subsequently, another conflict has arisen between the Amhara Popular Force (Fano) and Ethiopian government forces in Dabat town, disrupting its stability.

Consequently, Eritreans in this area confront a variety of challenges, including shortages in food, healthcare, and education, as well as having a higher risk of experiencing problems like rape, sexual abuse, witnessing the murder of family or friends, imprisonment, torching, and abduction. These collective challenges in the new environment may contribute to the development of symptoms such as PTSD, anxiety, and depression. These collective challenges in the new environment may contribute to the development of symptoms such as PTSD, anxiety, and depression. Currently, there is a lack of available data on these symptoms and their predictor variables in Dabat Town. Thus, the objective of this study was to assess the prevalence of PTSD, anxiety, and depression symptoms, along with associated factors, among Eritrean refugees in Dabat town, northwest Ethiopia. This will provide significant evidence for developing and implementing mental health intervention strategies that specifically address the particular difficulties faced by Eritrean refugees. Moreover, the study provides valuable insights that can guide the delivery of humanitarian assistance for refugees.

## Methods

### Study area, design and period

A community-based cross-sectional study was conducted from July 25 to September 30, 2023, at the Eritrea refugee camp in Dabat town, which is located in the Amhara regional state in northwest Ethiopia. The Dabat district is located about 775 km from Addis Ababa, the capital city of Ethiopia, and approximately 75 km from Gondar town. The camp was established to accommodate nearly 30,000 Eritreans displaced from the Tigray region, specifically from the Maiayni refugee camp, due to the conflict between the TPLF and the Ethiopian Federal Government forces. There are 30,000 Eritreans populated in one camp in Dabat town, northwest Ethiopia.

### Sample size determination and sampling technique

A total of 399 refugees were included in the study, based on an assumed proportion (p) of 37.8%, derived from a previous study conducted among Eritrean refugees at the Maiayni refugee camp [24].

$$ \text{n}\text{i}=\frac{{(\text{Z}{\upalpha }/2)}^{2}\text{*}\text{p}(1-\text{p})}{{\text{d}}^{2}}=\frac{{\left(1.96\right)}^{2}*0.378 (1-0.378)}{{\left(0.05\right)}^{2}}=362$$

With a 10% contingency rate, the final sample size was 399, Where; ni= initial sample size z α/2 = 1.96 (critical value for a normal distribution at a 95% confidence level), *p*= 0.46 (the proportion of stunting), d = 0.05 (the level of precision or acceptable error), Nf = final sample size.

A systematic random sampling method was employed to select a total of 193 male and 206 female Eritrean refugees.

### Variables

#### Dependent variables

Depression, Anxiety, and Posttraumatic Stress (Yes/No).

#### Independent variables

**Sociodemographic characteristics** (gender, age, monthly income, educational background), **behavioral factors** (khat chewing, cigarette smoking, exercise, alcohol consumption), and experiences of rape or sexual abuse, abduction, murder, and the availability of basic necessities like food, water, clothes, and shelter. **clinical and associated characteristics (**chronic diseases such as hypertension, Human immunodeficiency virus (HIV) and diabetes mellitus).

### Operational definition

#### Depression

In this study, depression was operationalized through participants’ scores, where individuals scoring ≥ 10 out of a potential 63 points were categorized as experiencing depression, whereas those scoring 0–9 were classified as not manifesting symptoms of depression [31].

#### Anxiety

Anxiety in this study was determined by participants scoring more than 7 out of 63 points, while those without anxiety were identified as individuals scoring 0–7 points [31].

#### Posttraumatic stress disorder

It was specified that study participants with a score of 14 out of 63 were categorized as having PTSD, whereas participants without PTSD were identified as those scoring 0–14 points [31].

### Data collection tool and procedure

Two skilled BSC nursing professionals collected the data using an interviewer-administered questionnaire. The questionnaire was translated from English into Tigrigna and back again by native speakers of both languages to ensure consistency and comprehensibility. The data collectors collected details on the educational background of the study participants by facilitating self-reporting through a questionnaire. The provided options in the questionnaire encompassed categories like “Never attended school,” “Primary School (Grade 1st–8th),” “Secondary School (Grade 9th–12th),” and “Diploma and above.” We used Lovibond’s short version of the DASS-21 (Depression, Anxiety, and Stress Scale-21), a psychological assessment tool designed to discern and distinguish symptoms associated with depression, anxiety, and stress [32–34]. Each of the three scales within the DASS-21 comprises seven items, which are further organized into subscales featuring similar content [32–34]. The threshold values for anxiety were categorized as follows: 0–7 (normal), 8–9 (mild), 10–14 (moderate), 15–19 (sever), and > 20 (extremely sever). The cut-off points for stress were delineated as 0–14 (normal), 15–18 (mild), 19–25 (moderate), 26–33 (sever), and > 34 (extremely sever ) [34].

Similarly for depression,0–9 (normal), mild [10–13], moderate [14–20], sever [21–27], ≥ 28 (extremely sever). The validity and reliability of the DAS-21 items have been tested and proven in previous studies conducted in Ethiopia [35].

### Data management and statistical analysis

Epidata version 4.6 was utilized for data entry, and subsequent analysis was conducted using SPSS version 25. Summary statistics, including proportions and frequencies, were employed to present the results in tables and graphs. The binary logistic regression model was applied to identify associated factors of posttraumatic stress, anxiety, and depression. Variables associated with having *p*-values (≤ 0.2) in the bivariable logistic regression model were included in a multivariable logistic regression model. Statistical significance in the multivariable binary logistic regression was determined by a *p*-value of (*p* ≤ 0.05), with the adjusted odds ratio used to determine the strength of association. The normality of continuous data was assessed using the Shapiro-Wilk test, and the model’s fitness was evaluated through the Hosmer-Lemeshow goodness-of-fit test. The validity of the questionnaire was examined using Cronbach’s alpha, revealing a satisfactory reliability coefficient of 0.635.

## Result

### Background characteristics of study participants

In this research, 399 participants were selected through a systematic random sampling technique, resulting in a 100% response rate. Approximately half of the participants were male, and the mean age of the study participants was 34 (± 0.54). Most of the research participants (66.4%) belonged to orthodox religious followers. Almost 50% of Eritrean refugees had jobs in the private sector. Of all the participants, 20.8% in the refugee camp have never attended formal education (Table 1).

**Table 1:** Background Characteristics of study participants in the Eritrea camp in Dabat town, northwest Ethiopia,2023 (*n* = 399)

Variables	Category	Frequency	Percentage
Sex	Male	193	48.4
	Female	206	51.6
Age(years)	< 24	21	5.3
	24–44	258	64.7
	≥ 44	120	30.1
Religion	Orthodox	265	66.4
	Muslim	90	22.6
	Catholic	45	8.8
	protestant	9	2.3
Employment statues	Employed	201	50.4
	Unemployed	198	49.6
Monthly income (ETB)	1000–2000	225	56.4
	2001–3500	92	23.1
	> 3500	82	20.6
Educational Status	Never attended school	83	20.8
	Primary School (Grade1^th−^8th )	112	28.1
	Secondary School (Grad 9th -12^th)^	189	47.4
	Diploma and above	15	3.8
Living Condition	With family	141	35.3
	Alone	258	64.7
Length of stayed at Camp	< years	110	27.6
	≥ 1years	289	72.4

### Clinical and behavioral characteristics of study participants

In this study, it was reported that over 50% of the participants had engaged in Khat chewing. Almost 47% of participants were cigarette smokers, and about 47% of the participants were cigarette smokers. Among the participants, one-fourth suffered from mental illness. Over 40% of participants indicated that there was a shortage of food or water in the refugee camps. More than one-third of the participants reported instances of friends or family being murdered, while 45% of participants experienced torture or beatings in the camp (Table 2).

**Table 2:** Clinical and behavioral characteristics of Study participants Eritrea camp in Dabat town, northwest Ethiopia,2023 (*n* = 399)

Variables	Category	Frequency	Percentage (%)
Khat chewing for the last three months	Yes	213	53.4
	No	186	46.6
Current Cigarette Smoking	Yes	180	47.3
	No	219	52.7
Participant’s psychiatry illness	Yes	102	25.6
	No	297	74.4
Family History psychiatry disorder	Yes	97	24.3
	No	307	75.7
Lack of food or water	Yes	162	40.6
	No	237	59.4
Lack of housing or closing	Yes	201	50.4
	No	198	49.4
Unnatural family death	Yes	103	25.8
	No	296	74.2
Combat situation	Yes	114	28.6
	No	285	71.4
Murder of family or friends	Yes	143	35.8
	No	256	64.2
Torched or beaten	Yes	182	45.6
	No	217	54.4
Imprisonment	Yes	233	58.4
	No	166	41.6
Being abducted	Yes	212	53.1
	No	187	46.9
Rape or sexual abused	Yes	101	25.3
	No	298	74.7
Chronic disease	Yes	191	48.1
	No	207	51.9
Types of chronic disease	Diabetic mellites	60	31.4
	Hypertension	90	47.1
	Heart disease	41	21.5

### Prevalence of symptoms of depression, anxiety and PTSD

The results of this study revealed that 45% (95% CI: 35.6-48.23), 33.6% (95% CI: 31.66–37.45), and 37.3% (95% CI: 35.56–40.34) of the participants had symptoms of depression, anxiety, and PTSD, respectively (Table 3).

**Table 3:** Prevalence of symptoms of depression, anxiety and PTSD among Eritrean refugees Camp in Dabat town, northwest Ethiopia,2023(*N* = 399)

Types of disorder	Classification	Frequency	Percentage (%)
Depression	Normal	219	55
	Mild	58	14.5
	Moderate	48	12
	Sever	11	2.8
	Total with Depression	180	45% (95% CI:39.6-48.23)
Anxiety	Normal	265	66.4
	Mild	66	16.5
	Moderate	53	13.3
	Sever	15	3.8
	Total with Anxiety	134	33.6% (95% CI:31.66–37.45)
PTSD	Normal	250	62.2
	Mild	50	12.5
	Moderate	38	9.5
	Sever	21	5.3
	Total with PTSD	149	37.34% (95% CI: 35.56–40.34)

### Factors associated with depression

In the bivariable analysis, variables such as sex, age, employment status, monthly income, education background, living conditions, incidents of rape or sexual abuse, lack of food or water, exposure to combat situations, the murder of family or friends, instances of being torched or beaten, abduction experiences, and imprisonment were considered as candidates for multivariable logistic regression (*p*-value ≤ 0.2). However, in the multivariable logistic regression analysis at a 95% confidence interval, variables such as sex, age, employment status, lack of food or water, being torched or beaten, and imprisonment were found to be statistically significant predictors of depression. Females had a 1.23 times higher chance of having depression compared to males (AOR = 1.23; 95% CI: 1.09–34*).* Study participants aged 45 years and older had a higher chance of developing depression as compared to their counterparts (AOR = 3.53; 95% CI: 1.09–7.67). Participants without jobs were more depressed as compared to those with jobs (AOR = 1.22; 95% CI: 1.08–3.87). Individuals who reported not having enough food or water have a higher chance of developing depression than those who did not report it (AOR = 1.23; 95% CI: 1.07–3.22). Finally, participants with a history of previous imprisonment were more likely to develop depression compared to those without any history of incarceration (AOR = 1.45; 95% CI: 1.09–3.76) (Table 4).

**Table 4:** Factors associated with depression among Eritrean refugees in Dabat town, northwest Ethiopia,2023 (*N* = 399)

Variables	Category	Depression		COR (95% CI)	AOR (95%CI
		Yes N (%)	No N (%)		
Sex	Male	103(52.3)	94(47.7)	1	1
	Female	86(42.8)	116(57.2)	1.47(1.19–2.88)	1.23(1.09-34) *
Age (years	< 24	28(62.2%)	17(37.8%)	1	1
	24–44	47(22.4)	12(77.6)	8.53(2.68–26.93)	1.81(0.98–5.76)
	≥ 45	105(72.4)	40(27.6)	13.63 (8.05–23.09)	3.53(1.09–7.67) *
Employment status	Employed	94(52.8)	84(47.2)	1	1
	Unemployed	86(38.7)	135(61.3)	1.75(1.08–4.59)	1.22(1.08–3.87*
Monthly income (ETB	< 1000	81(35)	151(65)	10.29(5.73–18.79)	4.52 (0.65–6.65)
	1000–2000	55(60)	37(40)	2.67 (1.45–4.94)	2.53(0.56–5.45)
	2001–3500	44(58.7)	31(41.3)	1	1
Education	Never attended school	47(45.6)	56(54.4)	0.75(0.22–0.58)	0.45(0.34–3.22)
	Primary school(grade1-8)	51 (38.6)	81(61.4)	0.56(0.28–0.97)	0.67(0.13–4.56)
	Secondar School (9–12)	82(61.2)	52(38.2)	1	1
Living condition	With family	98(51.3)	93(48.7)	1	1
	Alone	82(39.4)	126(60.6)	1.62(1.09–3.56)	1.23(0.89–4.56)
Rape or Sexual abuse	Yes	126(51.4)	119(48.6)	1.96(1.12–4.67)	1.3(1.09–3.66)
	No	54(34.6)	100(65.4)	1	1
Lack of food or water	Yes	86(65.1)	46(34.9)	5.05(1.06–7.56)	1.23(1.07–3.22) *
	No	64(27)	173(73)	1	1
Combat situation	Yes	159(53.7)	137(46.3)	4.53(1.09–6.20)	1.40(1.87–3.42)
	No	21(20.4)	82(79.6)	1	1
Murder of family or friends	Yes	119(72.5)	45(27.5)	7.54(4.95–12.28)	1.2(0.87–3.24)
	No	61(26)	174(74)	1	1
Torched or beaten	Yes	127(69.8)	55(30.2)	7.14(1.09–10.67)	1.34(1.13–4.56) *
	No	53(24.4)	164(75.6)	1	1
Being abducted	Yes	99(52.3)	90(47.7)	1.72 (0.55–4.35)	0.45(0.23–1.34)
	No	81(38.6)	129(61.4)	1	1
Imprisonment	Yes	143(61.3)	90(38.7)	5.53(1.15–9.87)	1.45(1.09–3.76)
	No	37(2.3)	129(77.7)	1	1

Hosmer and Lemeshow goodness-of-fit test *p*-value = 0.628

### Factors associated with anxiety

Employment status, murder of family or friends, being abducted, rape or sexual abuse, lack of food or water, being torched or beaten, and combat situation were considered as candidate variables of anxiety for multivariable analysis (*p* ≤ 0.2). Accordingly, employment status, murder of family or friends, rape or sexual abuse, being torched or beaten, and lack of housing or shelter were statistically significantly associated with anxiety at a *p* value of *p* ≤ 0.05. Compared to participants with jobs, those without jobs had higher odds of anxiety (AOR = 2.42; 95% CI: 1.19–3.22). Individuals in the study who witnessed the murder of friends or family members were more likely to have anxiety than those who did not witness such a murder (AOR = 1.32; 95% CI: 1.16–1.63). Study participants who experienced sexual abuse or rape had a higher likelihood of anxiety than those who did not (AOR = 1.20; 95% CI: 1.04–4.37). Individuals who experienced being torched or beaten have a higher level of anxiety compared to those who did not undergo such traumatic events (AOR = 1.26; 95% CI: 1.09–3.21). Lastly, individuals with reports of homelessness or shelter deficiency had a higher level of anxiety compared to those who did not report (AOR = 1.24; 95% CI: 1.04–6.33) (Table 5).

**Table 5:** Factors associated with anxiety among Eritrean refugees in Dabat town, northwest Ethiopia,2023 (*N* = 399)

Variables	Categories	Anxiety		COR 95%CI	AOR 95% CI
		Yes N (%)	No N (%)		
Employment status	Employed	89(44.3)	112(55.7)	1	1
	Unemployed	45(22.7)	153(77.3)	2.70(1.75–4.16)	2.42(1.19–3.22) *
Murder of family or friends	Yes	39(23.8)	125(76.2)	0.46(0.29–0.71)	1.32(1.16–4.63) *
	No	95(40.4)	140(59.6)	1	1
Being abducted	Yes	54(25.5)	158(74.5)	0.45(0.299–0.680	1.23(0.98–4.56)
	No	80(42.8)	107(57.2)	1	1
Rape or sexual abuse	Yes	32(31.7)	69(68.3)	1.21(1.06–1.34)	1.20(1.04–4.37) *
	No	102(34.2)	196(65.8)	1	1
Lack of food or water	Yes	56(34.6)	106(65.4)	1.07(0.70–1.64)	0.87(0.12–2.45)
	No	78(32.9)	159(67.1)	1	1
Torched or beaten	Yes	70(38.5)	112(61.5)	1.49(1.65–6.70)	1.26(1.09–3.21) *
	No	64(29.5)	153(70.5)	1	1
Combat situation	Yes	85(28.7)	211(71.3)	0.44(0.28–0.70)	0.23(0.12–0.66)
	No	49(47.6)	55(52.4)	1	1
Length of stayed at the Camp	< 1 year	24(21.8)	86(78.2)	0.45(0.72–0.75)	0.34(0.78–1.18)
	≥ 1 year	110(38.1)	179(61.9)	1	1
Lack of housing or Shelter	Yes	66(48.8)	78(51.2)	1.40(1.13–6.77)	1.24(1.06–4.33) *
	No	96(37.6)	159(62.4)	1	1
Current Khat Used	Yes	61(45.5)	73(54.5)	0.62(0.40–0.94)	1.63 (0.34–4.56)
	No	152(57.4)	113(42.6)	1	1
Current cigarette smoking	Yes	54(40.3)	80(59.7)	0.67 (0.44–1.20)	0.42(0.13–1.09)
	No	133(50.2)	132(49.8)	1	1
Social support	Poor	86(64.2)	48(35.8)	0.47(0.31–0.73)	1.22(0.45–1.34)
	Good	122(46)	143(54)	1	1

Hosmer and Lemeshow goodness-of-fit test *p*-value = 0.378

### Factors associated with PTSD

Variables like sex, age, employment status, murder of family or fringes, being abducted, rape or sexual abused, lack of food or water, Khat chewing, social support, length of stay at refugee camp, and lack of housing or shelter were candidate variables of PTSD for multivariable logistic regression (*p*-value ≤ 0.2). In the final model, compared to men, women had a higher chance of developing stress (AOR = 1.20; 95% CI: 1.07–4.31). Abducted individuals had higher odds of developing PTSD compared to those who were not abducted. (AOR = 1.42; 95% CI: 1.09–4.77). Participants who had suffered sexual abuse or rape had a higher chance of developing PTSD compared to those who had not (AOR = 1.30; 95% CI: 1.17–3.66). Individuals who indicated not having enough food or water had a higher chance of developing PTSD compared to those who did not report (AOR = 1.23; 95% CI: 1.07–4.22). Lastly, refugees who resided in refugee camps for one year or more exhibited a higher likelihood of experiencing PTSD **(**AOR = 2.63; 95% CI: 1.12–5.36) (Table 6).

**Table 6:** Factors associated with PTSD among Eritrean refugees in Dabat town, northwest Ethiopia,2023 (*N* = 399)

Variables	Category	Stress		COR	AOR
		Yes N (%)	No N (%)		
Sex	Male	46(47.9)	50(50.9)	1	1
	Female	103(34)	200(66)	1.78(1.09–3.15)	1.20(1.07–4.31) *
Age(years)	< 24	34(68)	16(32)	1	1
	24–44	40(17.5)	189(82.5)	4.36(0.80–6.92)	1.42(0.74–3.13)
	> 44	75(62.5)	45(37.5)	1	0.34(0.13–4.33)
Employment status	Employed	99(46)	116(54)	1	1
	Unemployed	50(27.17)	134(72.83)	2.28.07–4.35)	1.35(1.06–5.25) *
Murder of family or friends	Yes	76(41.8)	106(58.2)	1.41(0.72–1.82)	0.76(0.34-1.32)
	No	73(33.6)	144(66.4)	1	1
Being abducted	Yes	96(51.3)	91(48.7)	3.16(1.13–5.21)	1.42(1.09–4.77) *
	No	53(25)	159(75)	1	1
Rape or sexual abused	Yes	60(44.1)	76(55.9)	1.56(1.07–5.77)	1.30(1.17–3.66) *
	No	89(33.84)	174(66.16)	1	1
Lack of food or water	Yes	99(47.4)	110(52.6)	2.52(1.04–3.63)	1.23(1.07–4.220*
	No	50(26.3)	140(73.7)	1	1
Khat chewing	Yes	82(53.6)	71(46.4)	6.48(2.05–9.87)	1.32(0.07–4.17)
	No	67(27.2)	179(72.8)	1	1
Social support	Poor	108(51.7)	101(48.3)	3.88(2.35–7.87)	2.32(1.07–6.66)
	Good	41(21.6)	149(78.4)	1	1
Length of stayed at refugee Camp	≥ 1year	108(59.34)	74(40.66)	7.61(1.13–13.55)	2.63(1.12–5.36) *
	< 1year	41(16)	214(84)	1	1
Lack of housing or Shelter	Yes	102 (63)	60(37)	2.60(1.13–7.44)	2.20(1.09–5.32) *
	No	47(19.8)	190(80.2)	1	1

Hosmer and Lemeshow goodness-of-fit test *p*-value = 0.456

## Discussion

The objective of this study was to assess the prevalence of PTSD, anxiety, and depression symptoms, along with associated factors, among Eritrean refugees in Dabat town, northwest Ethiopia. The research revealed that the symptoms of anxiety, depression, and posttraumatic stress disorder (PTSD) were identified as 33.6% (95% CI: 31.66–37.45), 45% (95% CI: 39.6-48.23), and 37.3% (95% CI: 35.56–40.34), respectively. This research was similar to a study conducted among individuals living in a refugee camp in Greece, indicating a prevalence of 35.3% for PTSD, 33.3% for depression, and 27.9% for anxiety [36]. Nevertheless, the prevalence of PTSD in this study was found to be lower compared to studies conducted among Syrian refugees in Germany, 75.3% [37]; Southern Sudan (49.9%) [22]; and Eritrean refugees in Maiayni camp, Ethiopia,37.8% [24]. However, the prevalence of anxiety in this research was higher compared to that among Syrian refugees in Germany, which was reported to be 14% [37]. This variation could be attributed to factors such as the intensity of conflict, legal constraints, severe treatment by authorities, socioeconomic challenges, language barriers, discrimination, social isolation, restricted access to health services, and a lack of access to essential resources like nutritious food, clean water, and adequate clothing [37].

In this research, depression showed a positive correlation with variables such as sex, age, employment status, inadequate access to food or water, experiences of torture or physical abuse, a lack of housing or shelter, and instances of imprisonment. Female Eritreans were more dispersed than males. These findings are consistent with supporting evidence from the Greece refugee camp study [37]. This could be explained by biological factors, such as fluctuations in ovarian hormone levels, especially estrogen, which can cause alterations in mood that lead to anxiety and depression in women [38–41]. Study participants aged 45 years and older demonstrated an increased likelihood of developing depression compared to their counterparts. This study was consistent with a study done in Iraq [42] and in Ethiopia, Eritrean refugee camp [24]. This could be attributed to the physiological changes in both the cardiovascular and neurological systems that occur during the aging process, potentially increasing susceptibility to depression [43]. Refugees without employment had a higher chance of developing the odds of depression compared to their counterparts. This study was supported by a study conducted in Mexico [44] and Afghanistan [45]. The state of unemployment results in an inability to fulfill basic needs and cover essential expenses; these challenges significantly contribute to the onset of depressive symptoms [46, 47]. Refugees who reported a lack of food, water, shelter, and clothing in the refugee camp were found to have higher odds of experiencing depression compared to those who did not report such shortages. This study was similar to a study conducted in Uganda [48]. This could be due to insufficient food and water intake, which can lead to malnutrition and various health issues. Insufficient food and water intake can lead to malnutrition and various health issues. Malnutrition has been linked to changes in brain function and neurotransmitter imbalances, which can contribute to mood disorders, including depression [49, 50]. This can lead to a sense of isolation, shame, and a diminished identity, all of which can contribute to the development of depression [51]. Eritrean refugees who experienced torture or imprisonment were more likely to have depression than those who did not. This study was similar to a study conducted at Nyarugusu Refugee Camp in Kigoma, Tanzania [52]. This might be attributed to the fact that physical abuse or torture can lead to lasting physical injuries with potential long-term consequences. The presence of chronic pain or disability can contribute to depression as individuals contend with both the physical and emotional ramifications of their experiences [53]. Furthermore, studies suggest that unavoidable or uncontrollable stressors, like torture, can result in decreased dopamine release in the nucleus accumbens, leading to impaired responsiveness to environmental stimuli. This, in turn, may play a role in the onset and exacerbation of depressive symptoms [54].

The results of the current study showed a significant association between anxiety and a number of variables, such as employment status, instances of rape or sexual abuse, the death of family members or friends, experiences of sexual abuse, torture, and the lack of a house. This study was supported by a study conducted in refugee camps in Europe [55]. Individuals who watched or personally participated in the murder of a family member or friend were more likely to experience anxiety than those who had not. This study was consistent with one carried out at the Ethiopian refugee camp of Maiayni [24]. Experiencing the murder of family or friends is an intensely traumatic event for anyone, and for refugees, it can be especially devastating, leading to heightened levels of anxiety among them [56, 57]. This research also revealed that PTSD was positively associated with factors such as sex, age, employment status, length of stay at a refugee camp, experiences of abduction, rape, or sexual abuse, and the absence of food or water. This study was consistent with a study conducted in Darfur, Sudan [58], and Nepal [59].

Females were more likely to develop the odds of PTSD compared to males. This study is supported by a study done in Germany [60]. This could be due to the fact that women in refugee camps may face a higher risk of gender-based violence, including sexual assault and domestic violence. Such traumatic experiences can contribute significantly to the development of PTSD [60]. This could be due to the fact that women in refugee camps may face a higher risk of gender-based violence, including sexual assault and domestic violence. Such traumatic experiences can contribute significantly to the development of PTSD [61, 62]. The odds of having PTSD among older Eritrean refugees were higher than among younger refugees. This study was comparable to a study done by J. M. Hegeman et al. [63]. As older individuals often experience a higher prevalence of chronic health conditions, pain, and physical limitations, these persistent illnesses can ultimately contribute to the development of PTSD [42, 64]. Participants with a history of abduction, rape, or sexual abuse were more likely to have PTSD compared to those who did not have such a history. This study was in line with a study done in Mexico [44] and Sudan [22]. This could be attributed to the fact that refugees who have been the victims of sexual assault or kidnapping may suffer from miserable feelings of guilt and shame. These emotions have the potential to become internalized, which can result in a negative self-image and the emergence of variables that can exacerbate PTSD [22, 65]. Lastly, compared to refugees who stayed in the camp for less than a year, those who spent a year or more there had a higher chance of developing PTSD. This study was similar to one done with North Korean refugees [66], Iraq refugees [67], and Eritrean refugees in Ethiopia [24]. Living for an extended period of time in refugee environments can subject individuals to persistent stressors, including challenging living conditions, struggles to meet essential survival needs such as obtaining water, food, shelter, and healthcare, an inability to generate income, and isolation from family and traditional social support systems. The cumulative impact of these challenges increases the susceptibility of individuals to mental health issues, including PTSD [42, 68, 69].

### Limitation of the study

Due to the cross-sectional nature of the design, this study did not establish cause-and-effect relationships between our variables. Moreover, the study did not include data on the HIV status of participants because the study participants had not reported their HIV status during the data collection period.

## Conclusion and recommendation

The results of this study revealed that more than one-third of Eritreans living in the refugee camp in Dabat town had symptoms of PTSD, anxiety, and depression. This prevalence is higher than the previously reported studies. Several factors have been identified as contributing to the development of depression, anxiety, and post-traumatic stress disorder (PTSD). These factors included older age, gender (specifically being female), monthly income levels, unemployment status, experiences of rape or sexual abuse, witnessing the murder of family or friends, enduring physical harm such as torture or beatings, incarceration, and deprivation of fundamental needs such as food, shelter, and water. As a result, it is critical to prioritize early screening and intervention for post-migration mental health, particularly for women who have experienced traumatic events.

This research emphasizes the need for both governmental and non-governmental organizations to secure the provision of essential necessities such as food, clean water, shelter, clothing, and education. Additionally, this study calls for the legal protection of Eritrean refugees against arson, sexual abuse, rape, imprisonment without due process, and abduction. This research also highlights the need for healthcare service providers to implement a psychosocial intervention within the refugee community to enhance living conditions and address the effects of traumatic stressors. Furthermore, the concerned organization may then put these approaches into action to help minimize the occurrence of PTSD, anxiety, and depression symptoms among Eritrean refugees via early detection, prevention, and intervention. This study recommends using ordinal logistic regression to delve into a more comprehensive understanding of the severity levels associated with depression, anxiety, and PTSD in future research.

## Data Availability

No datasets were generated or analysed during the current study.

## Abbreviations

AOR: Adjusted Odds Ratio
CI: Confidence Interval
COR: Crude Odds Ratio
PTSD: Post-Traumatic Stress Disorder
SD: Standard Deviation
